# Did cybercrime cause the crime drop?

**DOI:** 10.1186/s40163-018-0082-8

**Published:** 2018-08-09

**Authors:** Graham Farrell, Daniel Birks

**Affiliations:** School of Law, University of Leeds, Leeds, LS2 9JT UK

**Keywords:** Cybercrime, Offender adaptation, Internet fraud, Crime drop, Crime decline, Security hypothesis, Displacement

## Abstract

Recent studies have hypothesised that the international crime drop was the result of the rise in cybercrime. We subject this ‘cybercrime hypothesis’ to critical assessment. We find significant evidence and argument indicating that cybercrime could not have caused the crime drop, and so we reject the cybercrime hypothesis.

## Introduction

Recent studies hypothesise that the long-term decline in crimes including burglary, car theft and assault, known as the international crime drop (van Dijk et al. 2012) were the result of increased cybercrime. We term this the ‘cybercrime hypothesis’. Four examples demonstrate:

First, Tcherni et al. (2016) conjecture that

*“A pronounced drop in crime, since the early 1990s, has encompassed every crime category … However, over the same period, the rates of online property crime (OPC) have been on the rise according to available evidence.”*

This study questions the existence of a property crime drop and suggest in relation to the violent crime drop that

*“some direct*-*contact predatory crimes may turn violent precisely because of the proximity of the offender and victim in time and space. This is not so true for cyber*-*predatory crimes since the two never meet in person. So, one indirect consequence of property crimes occurring online could be a drop in the violent crime rate.” (p. 907)*

Second, Caneppele and Aebi (2017) report that

*“the authors consider that the rise of online and hybrid crimes have contributed to the drop of offline crimes. This is a consequence of the development of the Internet …”. (p. 1)*

progressing to

*“The crime drop is an illusion then? The data presented in this paper suggest that a proper answer is ‘partially’.” (p. 10)*

Third, Button and Cross (2017) testify that

*“[Crime] over the last 20* *years has been falling and this has stimulated much debate over whether this is really happening… crime has shifted to new forms …[and] it is difficult to determine the true size of the [cyber*-*crime] problem and to what extent traditional criminals have moved to cyber frauds…”*

Fourth, Levi notes of economic cyber-crime that

*“it is not obvious whether this is just displacement for the fall in household and automobile property crime, nor how much overlap there is between the offenders and past ‘offline’ offenders.” (Levi* 2017 *; 3).*

There is some variation across authors in their emphasis, but their work informs the specification of a significant research question: Did cybercrime cause the crime drop? The remainder of this article examines the argument and evidence.

## Argument and evidence

### Lack of supporting evidence

Our initial observation is that the studies cited above fail to provide robust supporting evidence. They mostly assert that cybercrime rose concurrent with physical crime’s decline and suggest a causal connection. They justify a lack of analytic precision on the grounds that there is no firm evidence about cybercrime trends. However we suggest this misses the point because there is considerable evidence from which firm conclusions can be drawn, which becomes clear in what follows.

### Inconsistency in timings

Burglary and theft in the United States have declined since the early 1980s (Bureau of Justice Statistics 1994; Walters et al. 2013). From 1990, auto theft and violence began to fall in New York City (Bowling 1999), national trends shortly following suit (Truman and Planty 2012). Property crime in England and Wales peaked in 1992–1993, subsequently falling (ONS 2016). Operating under the assumption that cybercrime offenders require Internet access, initial adoption of the Internet is typically dated from the early nineties onward.^1^ The World Bank (2016) estimates the percentage of the population to have used the Internet (anywhere) in 1993 at 2% in the US and Australia and 0.5% in the UK—home access therefore being significantly lower. Comparing these pieces of evidence shows that the crime drop began before significant adoption of the Internet in the United States, England and Wales.

Property crime fell in Australia from around 2001 (Mayhew 2012), the closer temporal fit likely underpinning (Payne et al. 2018) interpretation that there has been some “displacement of crime and antisocial conduct into the online environment”. The US and UK evidence discussed above suggests any temporal correlation for Australia is spurious rather than causal. In addition, Brown (2015) observed that Australian offenders reported improved security as the most likely cause of the decline in their offending, while Kriven and Ziersch (2007) and others have provided more compelling alternative explanations for the decline in car theft. This point and the possibility of displacement are discussed further below.

### Cybercrime spread too slowly and in the wrong places

In addition to arriving too late, cybercrime spread too slowly to account for the first decade or more of the crime drop in the US and the UK. Indeed it is claimed that Internet adoption was highly unequal both within and between countries (e.g. Kiiski and Pohjola 2002). In most developed countries it began with several years of dial-up access, with bandwidth increasing over time. However, less affluent households more typically associated with offending are those gaining Internet access later than their affluent peers (Middleton and Sorensen 2006; Willis and Tranter 2006). It is also implausible that more rapidly increasing cybercrime in the twenty-first century had a causal effect, since adolescent offending rates were already decimated (Butts 2000, Farrell et al. 2015) and there was no acceleration in the rate of decline in physical crimes.

### Significant other evidence is inconsistent with cybercrime

Research has shown that declines in crime observed in Australia, Germany, England and Wales, the US and elsewhere are consistent with the introduction of improved security measures (Kriven and Ziersch 2007; Farrell et al. 2014). The cybercrime hypothesis, however, is inconsistent with significant components of the evidence. For example, increases in cybercrime cannot explain why, as vehicle crime fell in the UK and Australia, the age of stolen vehicles increased: the security hypothesis explains this as newer vehicles having better security. Likewise, cybercrime cannot explain how burglaries involving forced entry declined but unforced entries did not: the security hypothesis explains this as a result of security improvements at doors and windows. In fact the cybercrime hypothesis is inconsistent with virtually all the evidence relating to security that is tabulated by Tilley et al. (2018).

### It was not crime displacement or adaptation

The primary mechanism by which cybercrime is assumed to have induced the crime drop is attractive displacement: offenders substituting more attractive online crime for their physical crime. Yet for the most part it is difficult to see how cybercrimes serve as good substitutes. Cybercrime requires different physical and intellectual skills and experience, different tools, and mostly offers a different set of rewards under considerably different circumstances. Which cybercrime would be a good substitute for a street robbery, for instance? When the timing and diffusion of the Internet are considered, it is similarly implausible that teenagers skilled in joyriding adapted and switched to phishing, hacking or other cybercrimes and that this led to the decline in physical crime.

A more plausible scenario is adaptive switching for cyber-assisted crime such as fraud—if offenders recognised the opportunity and acquired the resources and skills. However, as discussed above, this would still occur too late to account for the decline in physical crimes.

Contrary to the crime drop, phone theft increased from the mid-1990s and gained renewed momentum with the spread of smartphones from the late noughties (Harrington and Mayhew 2001; Farrell 2015). If displacement or adaptation to cybercrime caused most street crimes to decline, why not phone theft too?

### More cybercrime, less violence?

Tcherni et al. propose that online crime is less violent and so violence declined. It is plausible that online crime is less likely, on average, to result in violence. However, the suggestion that there is a causal connection is misplaced: it involves the same mistakes with regard to the timing and displacement/adaptaton that were discussed above.

### It was not Internet-induced lifestyle or cultural change

It is plausible that the Internet has changed lifestyles and culture—keeping potential offenders and victims indoors thereby reducing their involvement with contact crime and increasing guardianship of property. While arguably a distinct hypothesis, it warrants mention here. It is subject to the timing critique discussed above. Other lifestyle changes also do not fit: reductions in alcohol use by adolescents in England, for example, occurred from 2003, which is a decade later than the crime drop (Bhattacharya 2016). It is also conceivable that security technologies mean adolescents stay home more because joyriding and other physical crimes are less attractive.

## Conclusion

Cybercrime did not cause the crime drop. The timing is wrong. The causal mechanisms are wrong. There is no robust evidence supporting, but a wealth of evidence inconsistent with, the cybercrime hypothesis. There are too few decent measures of cybercrime but this is no reason to notionally construct a major trend and claim it caused a drop in physical crimes internationally.

We suggest that the crime drop and rising cybercrime are independent trends caused by broad changes to crime opportunity structures. Opportunities for physical crime declined due to security improvements. Opportunities for cybercrime increased as the Internet spread.

## Footnotes

1 Technically, Tim Berners Lee made the World Wide Web available on 6 August 1991, the first image was uploaded in 1992, the Mosaic browser was launched in late 1993, Netscape was founded in 1994 and went on sale in 1995. The internet was publicly launched by AOL in 1994 and various indicators suggest it really started to spread in the US from 1995.
